# Long-term outcomes of endoscopic submucosal dissection for high-grade dysplasia and early-stage carcinoma in the colorectum

**DOI:** 10.1186/s40880-018-0273-4

**Published:** 2018-03-21

**Authors:** Tao Chen, Wen-Zheng Qin, Li-Qing Yao, Yun-Shi Zhong, Yi-Qun Zhang, Wei-Feng Chen, Jian-Wei Hu, Marie Ooi, Ling-Li Chen, Ying-Yong Hou, Mei-Dong Xu, Ping-Hong Zhou

**Affiliations:** 1 Endoscopy Center, Zhongshan Hospital and Endoscopy Research Institute, Fudan University, 180 Fenglin Road, Shanghai, 200032 P. R. China; 2 Department of Pathology, Zhongshan Hospital, Fudan University, Shanghai, 200032 P. R. China

**Keywords:** Early-stage carcinoma, High-grade dysplasia, Colorectum, Endoscopic submucosal resection

## Abstract

**Background:**

Colorectal carcinomas (CRCs) arise from premalignant precursors in an adenoma-carcinoma sequence, in which adenoma with high-grade dysplasia (HGD) and early-stage carcinoma are defined as advanced neoplasia. A limited number of studies have evaluated the long-term outcomes of endoscopic submucosal dissection (ESD) for advanced colorectal neoplasia. This study aimed to assess the efficacy and safety of ESD for advanced colorectal neoplasia as well as the long-term outcomes, including local recurrence and metastasis.

**Methods:**

We analyzed data collected from 610 consecutive patients with 616 advanced colorectal neoplasia lesions treated with ESD between January 2007 and December 2013. Clinical, endoscopic, and histological data were collected over a median follow-up period of 58 months to determine tumor stage and type, resection status, complications, tumor recurrence, and distant metastasis.

**Results:**

The overall rates of en bloc resection, histological complete resection, and major complications were 94.3%, 89.4%, and 2.3%, respectively. Hybrid ESD was an independent factor of piecemeal resection. Tumor location in the colon was associated with increased risk of ESD-related complications. During the follow-up period, all patients remained free of metastasis. However, local recurrence occurred in 4 patients (0.8%); piecemeal resection was a risk factor.

**Conclusions:**

ESD is effective and safe for resection of advanced colorectal neoplasia, with a high en bloc resection rate and favorable long-term outcomes. ESD is indicated for the treatment of HGD and early-stage CRC to obtain curative resection and reduce local recurrence rate.

## Background

Colorectal carcinoma (CRC) is currently a common cancer and is the third leading cause of cancer-related death worldwide [1, 2]. Most CRCs arise from premalignant precursors along a long-term adenoma-carcinoma sequence [3]. In this sequence, adenoma with high-grade dysplasia (HGD) and early-stage carcinoma are defined as advanced neoplasia [4, 5]. Identification and removal of such early-stage tumors have been associated with the prevention of CRC-related death [6]. Recently, endoscopic submucosal dissection (ESD) has been increasingly used for colorectal epithelial lesions [7]. Compared with conventional endoscopic resection, ESD enables en bloc resection with a high rate, which contributes to accurate histological evaluation [8, 9]. The number of studies on outcomes of ESD for superficial colorectal tumors is increasing [7, 10, 11]. However, data on the long-term outcomes of ESD for advanced colorectal neoplasia are still lacking. Hence, this study was performed to assess the efficacy, safety, and long-term outcomes of ESD for advanced colorectal neoplasia.

## Patients and methods

### Patient selection

ESD was initially indicated for lesions requiring endoscopic en bloc resection, for which the snare technique is difficult to implement, including laterally spreading tumors (LSTs), superficial invasive submucosal carcinoma, large depressed tumors, and large elevated lesions that are likely early-stage cancers [10, 12]. Consecutive patients with advanced colorectal neoplasia treated with ESD at our center between January 2007 and December 2013 were selected. Exclusion criteria included findings of a deep submucosal invasive carcinoma, as determined by endoscopic examination, and the presence of other invasive carcinomas and circumferential tumors that required surgical resection because of the increased technical difficulty and the anticipated risk of stenosis.

The clinicopathologic information of selected patients was collected to analyze the long-term outcomes after ESD. The study was performed in accordance with the Declaration of Helsinki (5th version). All patients were informed of the risks and benefits of ESD, and each patient provided written informed consent for the procedure. This study protocol was approved by the institutional review board of Zhongshan Hospital, Fudan University (No. 2009135).

### Endoscopic submucosal dissection

**Fig. 1:**
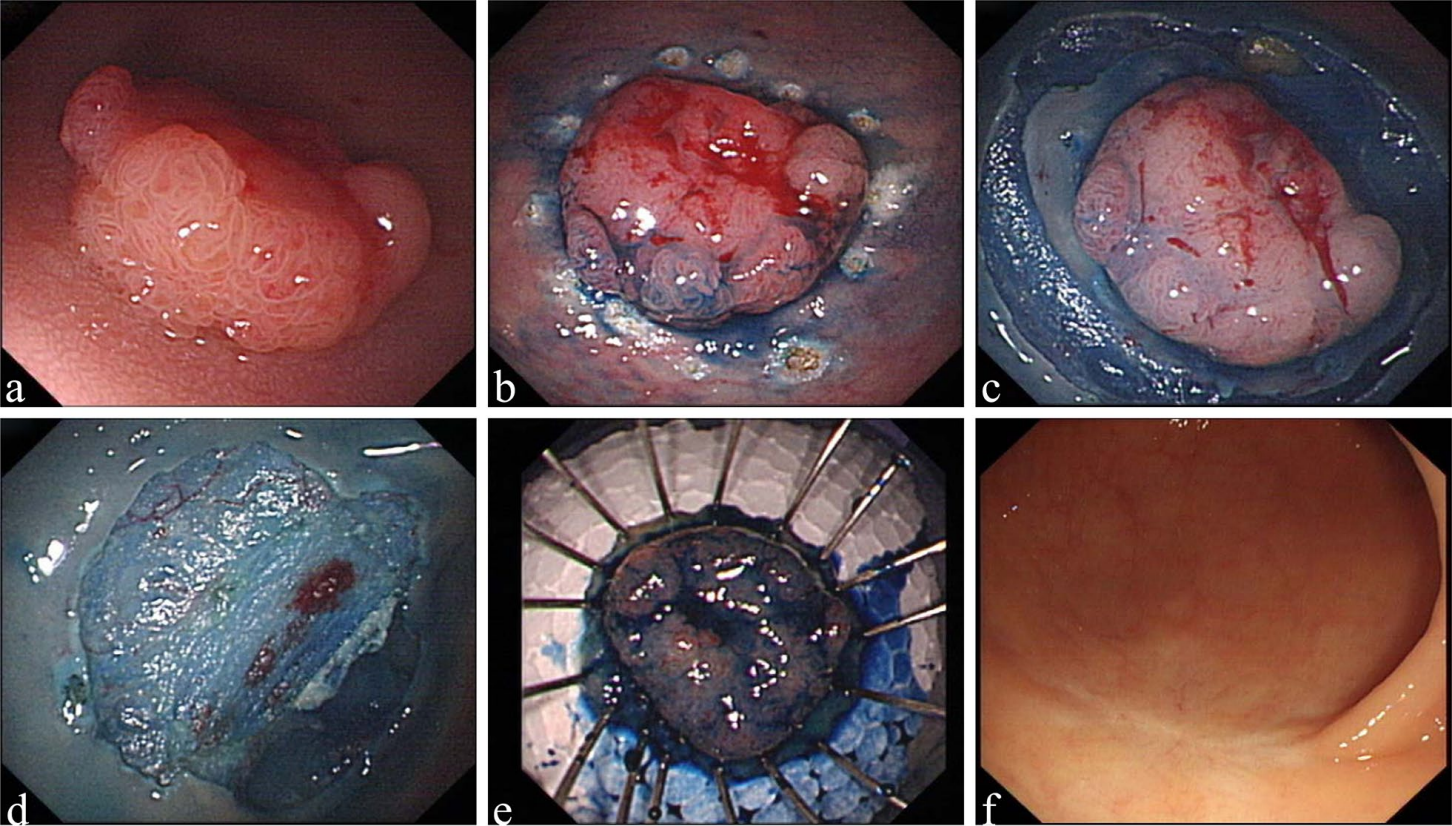
Endoscopic submucosal dissection (ESD) of a high-grade rectal dysplasia. **a** Endoscopic view of the lesion; **b** marker dots on the normal mucosa; **c** mucosal incision along the marker dots; **d** the artificial ulcer bed after ESD; **e** tissue specimens fixed to a wooden plate using thin needles; **f** endoscopic view of the scar during follow-up period

ESD was performed with the patient under general intravenous anesthesia, and indices of the patient’s cardiorespiratory functions (heart rate, blood pressure, and oxygen saturation) were continually monitored. Endoscopic equipment and accessories were introduced as described in previous reports [13, 14]. The procedure began by marking dots on the normal mucosa approximately 5 mm away from the tumor (Fig. 1). After the submucosal injection of 5 mL of solution (including 5% indigo carmine and 1% epinephrine) using a 23-gauge disposable needle, a mucosal incision was created along the marker dots using a needle knife. Next, under direct vision, we continued to use a hook knife to dissect the submucosal connective tissue beneath the tumor; the muscularis propria layer along the edge of the lesions could also be peeled if necessary. During the dissection, the solution was injected repeatedly if necessary. After resection was completed, all visible vessels of the artificial ulcer bed were thoroughly coagulated with argon plasma coagulation to prevent postoperative bleeding. Hybrid ESD was defined as the combined use of conventional endoscopic resection and the ESD technique, with a special ESD knife, and resection was completed by snaring [7, 12, 15]. Hybrid ESD was performed when standard ESD was infeasible.

After ESD, surgery was recommended when tumors were suspicious with risk factors of lymph node metastasis such as deep submucosal invasion, lymphovascular infiltration, poor differentiation, and tumor budding.

### Histopathological examination

Resected specimens were stretched, pinned, fixed in formalin solution and assessed microscopically. The World Health Organization classification for tumors of the digestive system was used for histopathological evaluation [16]. An en bloc resection was defined as an excision of the tumor in one piece without fragmentation. Histological complete resection was defined as en bloc resection with negative horizontal and vertical margins. The definition of R0 resection was a histological complete resection and no risk of lymph node metastasis according to histological examination of the resected specimen following the Japanese Society for Cancer of the Colon and Rectum (JSCCR) guideline criteria [17].

### Complication observation

Bleeding and perforation are major ESD-related complications. Minor bleeding was treated with immediate coagulation and was not viewed as a complication. Delayed bleeding was defined as hematochezia or melena requiring an endoscopic hemostatic procedure from 0 to 14 days after ESD completion.

### Follow-up

All patients were followed up either at our institution or in partnership with their referring centers. Follow-up colonoscopy was performed at 6 and 12 months after colorectal ESD. Then, patients were suggested to be followed according to the surveillance guidelines [18, 19]. The last follow-up was performed in December 2016.

### Data collection and statistical analysis

Clinicopathological and endoscopic data, including age, sex, tumor characteristics, histology, resection status, complications, local recurrence, and metastasis, were collected from the database of colorectal ESDs of our center and analyzed. Statistical analysis among groups was performed using Pearson’s Chi square test, a continuity correction Chi square test, or Fisher’s exact test as appropriate. Risks for piecemeal resection, complications, and local recurrence were assessed by univariate and/or multivariate logistic regression analyses as appropriate. SPSS Statistics 18 software (IBM, Chicago, IL, USA) was used for statistical analysis. *P *< 0.05 was considered to be statistically significant.

## Results

### Characteristics of patients

**Fig. 2:**
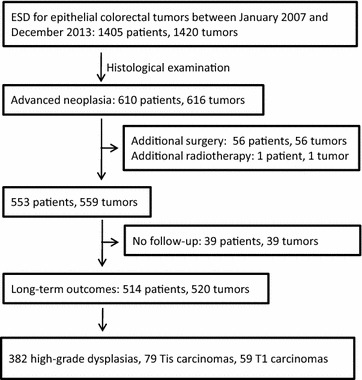
Flowchart of selecting patients with high-grade dysplasia and early-stage carcinoma of the colorectum who underwent ESD

Between January 2007 and December 2013, 610 patients (354 men and 256 women) with 616 advanced colorectal neoplasia lesions were identified and analyzed in this study (Fig. 2). Six patients had 2 lesions. The median age of the patients was 64 (range, 23–89) years; 370 (60.7%) patients were older than 60 years.

### Pathologic results

The average tumor size was 2.9 ± 1.3 cm (median, 2.5 cm; range, 1.0–9.0 cm). As shown in Table 1, polypoid tumor was the most common macroscopic type, and HGD was the most common histological type. Only 12 (1.9%) lesions presented with invasive lymphovascular infiltration.

**Table 1:** Baseline characteristics of 616 advanced colorectal neoplasia lesions in 610 patients

Variable	Number of lesions (%)
Tumor size (cm)	
< 4.0	483 (78.4)
≥ 4.0	133 (21.6)
Tumor location	
Right side of the colon	110 (17.8)
Left side of the colon	142 (23.1)
Rectum	364 (59.1)
Growth type	
LST-G	174 (28.2)
LST-NG	118 (19.2)
Polypoid	324 (52.6)
Histology	
HGD	391 (63.5)
Carcinoma	
Tis	98 (15.9)
T1a	80 (13.0)
T1b	47 (7.6)
Lymphovascular infiltration	
Absence	604 (98.1)
Presence	12 (1.9)

*LST* laterally spreading tumor, *LST-G* granular LST, *LST-NG* non-granular LST, *HGD* high-grade dysplasia, *Tis* carcinoma in situ, *T1a* carcinoma with submucosal invasion < 1000 μm in depth, *T1b* carcinoma with submucosal invasion ≥ 1000 μm in depth

A total of 57 patients had deep submucosal invasion and/or invasive lymphovascular infiltration: 56 accepted additional radical surgery, and 1 with rectal lesion rejected additional radical surgery and underwent additional local radiotherapy. Two patients with T1b carcinomas (submucosal invasion ≥ 1000 μm in depth) had a histological complete resection. Although additional surgery was recommended to the 2 abovementioned patients, it was rejected because of the high surgical risk associated with comorbidities and old age, and instead each accepted an intensive follow-up.

### Feasibility of endoscopic submucosal dissection

En bloc resection was achieved in 581 (94.3%) of the 616 lesions, and histological complete resection was achieved in 551 (89.4%) lesions. Outcomes related to ESD are shown in Table 2. Univariate analysis showed that tumor location and resection method were significantly associated with the ESD piecemeal resection rate. Multivariate analysis indicated that piecemeal resection rate was independently higher in tumors treated by hybrid ESD than in tumors treated by standard ESD (Table 3).

**Table 2:** Outcomes related to ESD for advanced colorectal neoplasia

Variable	Number of lesions (%)
Short-term outcomes	
Resection status	
En bloc	581 (94.3)
Piecemeal	35 (5.7)
Histological complete resection	
Complete	551 (89.4)
Incomplete	65 (10.6)
Complications	
Postoperative bleeding	10 (1.6)
Intraoperative perforation	4 (0.6)
Long-term outcomes	
Recurrence	4 (0.8)
Metastasis	0 (0.0)

*ESD* endoscopic submucosal dissection

**Table 3:** Univariate and multivariate logistic regression analyses on the associations between clinicopathological characteristics of patients with advanced colorectal neoplasia and piecemeal resection by ESD

Variable	Total (number of patients/lesions)	Univariate analysis		Multivariate analysis	
		OR (95% CI)	*P* value	OR (95% CI)	*P* value
Age (years)					
≤ 60	238	1.000		1.000	
> 60	372	0.698 (0.355–1.372)	0.295	0.714 (0.347–1.469)	0.360
Sex					
Male	357	1.000		1.000	
Female	253	0.858 (0.430–1.710)	0.663	0.842 (0.408–1.739)	0.642
Tumor size (mm)					
< 40	483	1.000		1.000	
≥ 40	133	1.238 (0.568–2.702)	0.590	1.032 (0.445–2.389)	0.942
Tumor location					
Rectum	364	1.000		1.000	
Colon	252	2.342 (1.182–4.639)	0.012	2.058 (0.997–4.248)	0.051
Growth type					
Polypoid + LST-NG	442	1.000		1.000	
LST-G	174	1.290 (0.630–2.641)	0.485	2.179 (0.981–4.840)	0.056
Histology					
HGD + Tis	489	1.000		1.000	
T1a + T1b	127	1.324 (0.497–3.527)	0.773	1.693 (0.588–4.870)	0.329
Resection method					
Standard ESD	529	1.000		1.000	
Hybrid ESD	87	7.406 (3.678–14.914)	< 0.001	8.123 (3.820–17.272)	< 0.001

*ESD* endoscopic submucosal dissection, *OR* odds ratio, *CI* confidence interval, *LST* laterally spreading tumor, *LST-G* granular LST, *LST-NG* non-granular LST, *HGD* high-grade dysplasia

### Complications

Ten (1.6%) patients who underwent endoscopic hemostatic procedures had postoperative bleeding, and 4 (0.7%) patients had a perforation during the ESD procedure (Table 2). All cases of recognized intraoperative perforation or postoperative bleeding were successfully managed by endoscopic closure or coagulation. Perforations were also managed with subsequent conservative treatment, including fasting and intravenous antibiotics for a few days. There were no treatment-related deaths. In both the univariate and multivariate analyses, tumor location in the colon was an independent contributor to ESD-related complications (Table 4).

**Table 4:** Univariate and multivariate logistic regression analyses on the association between clinicopathological characteristics of patients with advanced colorectal neoplasia and ESD-related complications

Variable	Univariate analysis		Multivariate analysis	
	OR (95% CI)	*P* value	OR (95% CI)	*P* value
Age (years)				
≤ 60	1.000		1.000	
> 60	0.909 (0.341–2.423)	0.849	0.863 (0.316–2.357)	0.774
Sex				
Male	1.000		1.000	
Female	0.951 (0.357–2.532)	0.920	0.984 (0.363–2.670)	0.975
Tumor size (mm)				
< 40	1.000		1.000	
≥ 40	1.549 (0.536–4.476)	0.607	1.769 (0.587–5.329)	0.311
Tumor location				
Rectum	1.000		1.000	
Colon	3.961 (1.377–11.391)	0.006	4.212 (1.437–12.344)	0.009
Growth type				
Polypoid + LST-NG	1.000		1.000	
LST-G	0.776 (0.250–2.415)	0.869	0.757 (0.236–2.427)	0.639
Histology				
HGD + Tis	1.000		1.000	
T1a + T1b	0.886 (0.861–0.912)	0.280	0.000 (0.000)	0.997
Resection method				
Standard ESD	1.000		1.000	
Hybrid ESD	0.806 (0.181–3.589)	0.777	0.556 (0.120–2.577)	0.453

*ESD* endoscopic submucosal dissection, *OR* odds ratio, *CI* confidence interval, *LST* laterally spreading tumor, *LST-G* granular LST, *LST-NG* non-granular LST, *HGD* high-grade dysplasia

### Follow-up results

Excluding the 57 patients who underwent additional surgery or radiotherapy, 533 patients were evaluated for the outcomes of ESD. Thirty-nine patients were lost to follow-up; among them, 27 had HGD, 9 had carcinomas in situ, and 3 had T1a carcinomas (submucosal invasion < 1000 μm in depth). The other 514 patients with 520 lesions had a median follow-up period of 58 months (range, 36–117 months). Local recurrence was detected in 4 (0.8%) patients, all of whom showed HGD (Table 2). Piecemeal resection was a significant contributor to local recurrence (Table 5). No patients developed metastasis to either the lymph nodes or distant organs during the follow-up period.

**Table 5:** The association between clinicopathological characteristics of 514 patients with 520 advanced colorectal neoplasia lesions and local recurrence after ESD

Variable	Total (number of patients/lesions)	Local recurrence (%)	*P* value
Age (years)			
≤ 60	202	1 (0.5)	0.943
> 60	312	3 (1.0)	
Sex			
Male	291	4 (1.4)	0.136
Female	223	0 (0.0)	
Tumor size (mm)			
< 40	412	2 (0.5)	0.408
≥ 40	108	2 (1.9)	
Tumor location			
Rectum	328	1 (0.3)	0.287
Colon	192	3 (1.6)	
Growth type			
Polypoid + LST-NG	373	2 (0.5)	0.681
LST-G	147	2 (1.4)	
Histology			
HGD + Tis	461	4 (0.9)	1.000
T1a + T1b	59	0 (0.0)	
Lymphovascular infiltration			
Absence	515	4 (0.8)	1.000
Presence	5	0 (0.0)	
Resection status			
En bloc	491	2 (0.4)	0.005
Piecemeal	29	2 (6.9)	
Resection method			
Standard ESD	449	2 (0.4)	0.163
Hybrid ESD	71	2 (2.8)	

*ESD* endoscopic submucosal dissection, *LST* laterally spreading tumor, *LST-G* granular LST, *LST-NG* non-granular LST, *HGD* high-grade dysplasia

## Discussion

In the present study, ESD achieved en bloc resection and histological complete resection rates of 94.3% and 89.4% for patients with advanced colorectal neoplasia, and the rate of major complications was only 2.3%. Hybrid ESD was an independent factor of piecemeal resection. Tumor location in the colon was associated with increased risk of ESD-related complications.

ESD offered a high en bloc and complete resection rate for early-stage CRC and HGD in the colorectum, even for lesions that were larger than 4.0 cm in diameter or located deeper in the submucosal layer [8, 9]. Additionally, the complete resection rate of ESD was relatively higher than that of endoscopic mucosal resection (EMR) [8]. On the other hand, EMR is unlikely to offer a high complete resection rate for large colorectal epithelial lesions or for post-EMR tumor recurrences. A piecemeal EMR resection results in increased post-EMR recurrences and uncertainty in histological assessment of the complete resection [20]. However, ESD of colorectal tumors is technically difficult. Therefore, hybrid ESD (a combined EMR and ESD technique) has been proposed to facilitate the ESD procedure and could be an option in certain situations [21, 22]. The multivariate analysis conducted in the present study indicated that the piecemeal resection rate was independently higher for tumors treated with hybrid ESD than for tumors treated with standard ESD. The risk of recurrence after piecemeal removal is relatively high, suggesting that standard ESD is preferred for patients with potential malignance [3].

Previous work indicated that ESD was the first-line treatment for tumors larger than 2.0 cm [23]. In the present study, ESD was indicated for lesions requiring endoscopic en bloc resection for which it was difficult to use the snare technique. ESD was the preferred treatment for advanced colorectal neoplasia and tumors with higher malignant potential, such as non-granular LST (LST-NG) [24], irrespective of tumor size. Notably, tumor location in the colon contributed to piecemeal resection in the present study. Hayashi et al. [25] indicated that poor endoscopic operability was an independent predictor of histological incomplete resection and perforation. Hori et al. [26] studied predictive factors for technical difficulty in ESD of the colorectum and indicated that colon flexure location was an independent risk factor for piecemeal resection. Isomoto et al. [27] reported that a tumor located in the right-side colon was an independent risk factor for histological incomplete resection. Although multivariate analysis indicated that tumor location was not an independent risk factor of piecemeal resection in the present study, ESD for colon tumors requires more experience and attention due to the unique anatomical characteristics of the colon and its flexures.

Repici et al. [28] suggested that colorectal anatomic characteristics were the main contributors to the risk of ESD-related complications. We noted that colon tumor location was an independent risk factor for complications. Hori et al. [26] also indicated that tumor location at the colon flexure was an independent risk factor for ESD-related complications and that ESD for colon tumors requires more technical skills and, especially for tumors located at flexures. However, the importance of tumor location was influenced by the endoscopist’s experience.

Our results indicate an adequate safety profile for colorectal ESD. Major complications occurred in 14 (2.3%) patients: 10 had postoperative bleeding, and 4 had a perforation. Postoperative bleeding could be managed with endoscopy without emergency surgery, and patients then often recovered under conservative observation. In the present study, all cases of recognized perforation were successfully managed immediately by endoscopic closure using endoclips.

In the present large consecutive study with a median follow-up period of more than 4 years, neither ESD-related nor disease-specific death was observed. The overall local recurrence rate was low and occurred in patients with piecemeal resection of tumors. As discussed above, hybrid ESD is a significant and independent contributor to piecemeal resection, which is the most important risk factor for local recurrence after endoscopic resection for colorectal neoplasia [7]. Consistently, in the present study, we found that the local recurrence rate of patients treated with hybrid ESD was higher than that of patients treated with standard ESD, but without significant difference, probably because the proportion of patients who underwent hybrid ESD was low. Thus, standard ESD is indicated for treating carcinomatous lesions because it features en bloc resection, which may decrease the risk of local recurrence.

The potential malignance of a lesion should be considered before choosing the endoscopic resection method. A regular endoscopic examination during follow-up is important, particularly for patients with piecemeal resection. Follow-up colonoscopy was recommended at 3–6 months after piecemeal resection, and it has been reported that most local recurrent lesions were detected within 12 months after the initial endoscopic resection [28, 29]. When local recurrence occurs, additional ESD was acceptable [30, 31].

For patients with invasive tumors, such as T1 carcinomas with deep submucosal invasion, surgical treatment is safer than ESD. It was reported that long-term outcomes were favorable if patients with non-R0 resection undergo appropriate additional surgical resection [10]. According to the 2014 JSCCR Guidelines for the Treatment of Colorectal Cancer [17], patients with T1 CRC should be considered for additional colectomy with lymph node dissection. However, the probability of lymph node metastasis is extremely low if no other risk factors exist [10, 32, 33]. In the present study, 2 patients with T1 CRC had a histological complete resection. Although additional surgery was recommended, it was rejected by these 2 patients because of high surgical risk, and instead each patient accepted an intensive follow-up. No recurrence or lymph node metastasis occurred during the follow-up period.

The limitations of the present study included its retrospective design and single-center analysis, although we evaluated many consecutive case series. Thus, a prospective and multi-center study is warranted.

## Conclusions

ESD is effective and safe for the resection of advanced colorectal neoplasia, with a high en bloc resection rate and favorable long-term outcomes. ESD is indicated for the treatment of early-stage CRC and HGD to obtain curative resection and prevent local recurrence.
